# Effectiveness of citrulline and N-carbamoyl glutamate as arginine precursors on reproductive performance in mammals: A systematic review

**DOI:** 10.1371/journal.pone.0209569

**Published:** 2018-12-20

**Authors:** Jorge Y. P. Palencia, Alysson Saraiva, Márvio Lobão Teixeira Abreu, Marcio G. Zangeronimo, Allan P. Schinckel, Cesar Augusto Pospissil Garbossa

**Affiliations:** 1 Department of Animal Science, Universidade Federal de Lavras, Lavras, Minas Gerais, Brazil; 2 Department of Animal Science, Universidade Federal de Viçosa, Viçosa, Minas Gerais, Brazil; 3 Department of Veterinary Medicine, Universidade Federal de Lavras, Lavras, Minas Gerais, Brazil; 4 Animal Science Department, Purdue University, West Lafayette, Indiana, United States of America; 5 Department of Animal Nutrition and Production, School of Veterinary Medicine and Animal Sciences, University of São Paulo, Pirassununga, São Paulo, Brazil; University Medical Center Utrecht, NETHERLANDS

## Abstract

The use of functional nutrients has been proposed to reduce the occurrence of intrauterine growth retardation in animals at birth in several mammalian species. The objective of this study was to verify the effectiveness of citrulline and N-carbamylglutamate (NCG) dietary supplementation as arginine precursors for mammalian species, and the effects on fetal development through a systematic review. The search for studies was performed during August 2018 in the PubMed, ISI Web of Science, Science Direct, and Scopus databases. The literature search was conducted using “arginine precursor”, “citrulline”, or “N-carbamylglutamate” as keywords, combined with “gestation”, “pregnancy”, “fetus”, “newborn”, or “reproduction”. Studies in which arginine precursors were evaluated in gestating mammals and their effects on parameters related to the intrauterine development of the conceptus were selected. Of 1,379 articles, 18 were selected, primarily based on the title and the abstract. Supplementation with NCG (0.5 g to 2 g/kg of feed) increased maternal plasma arginine concentrations in all studies that evaluated this variable. Fetal number increased in 55.56% of the studies that evaluated it, and fetal weight increased in the majority (62.5%) of the studies evaluating this variable. By supplementing citrulline, only fetal weight was improved, with an increase in maternal plasma arginine in 40% of the studies. In conclusion, N-carbamoyl glutamate seems to be an arginine precursor more effective than L-citrulline during gestation; however, both precursors, beside L-Arginine, should be evaluated in similar conditions to confirm the existence of specific particularities such as periods and levels of supplementation, which need to be considered for different species of animals. The supplementation of NCG increases arginine concentrations in maternal plasma, thus improving mammalian reproductive efficiency and fetal development, mainly by promoting higher birth weight.

## Introduction

Lower fetal numbers and reduced fetal growth are the result of an imbalance between the placenta’s ability to deliver oxygen and/or nutrients and fetal demand [1]. It is a major problem associated with livestock production because of increased rates of perinatal morbidity and mortality, increased incidence of disease, reduced productive performance, reduced carcass and meat quality, and even reduced performance in animal athletes [2; 3].

Gestational nutrition affects the health and development of the progeny through the epigenetic pathway by altering mRNA expression for genes involved in morphological, organogenic, and adaptive physiological responses. This phenomenon is known as “fetal programming through nutrition” [4].

Dietary supplementation with L-arginine during gestation improves fetal development due to the participation of this amino acid in metabolic pathways of the formation of active molecules, such as polyamines and nitric oxide [5]. This process contributes to implantation, embryogenesis, uterine quiescence during gestation, growth, development, and fetal survival [6; 7; 8].

However, the biological half-life of ARG (arginine) in mammals is relatively short, with 45 min in sheep and sows at 105 days of gestation [9; 10]. This occurs due to the high arginase activity, rapidly degrading ARG in tissues [11; 12; 13]. Moreover, the supply of ARG via ruminant feed leads to rumen degradation, which necessitates the use of protected ARG, which has a greater cost than normal ARG [14]. Furthermore, Zhang et al. [15] state that because of the high cost of ARG, it is currently economically unfeasible in pig production. Furthermore, ARG and lysine are basic amino acids and compete for the same transport system [16]. Therefore, high dietary levels of ARG might cause competition for transporters, hindering lysine absorption [17; 18]. Thus, the use of arginine precursors, such as N-carbamoylglutamate (NCG) and L-citrulline (CIT), may be a more viable alternative to obtain the benefits provided by arginine. Also, these additives are not extensively degraded in the rumen [19; 20].

The CIT molecule is one of the endogenous precursors of ARG through its conversion by argininosuccinate synthase and lyase. Also, CIT provides substrate for various metabolic molecules including polyamines and nitric oxide synthase (NOS) to produce NO [21]. Overall, CIT is more effective than ARG in increasing NO concentrations due to its lower hepatic catabolism and higher bioavailability when provided to animals [22; 23]. Furthermore, CIT undergoes little degradation in the placenta, thus allowing its maximum transfer from the mother to the fetus [24; 25] and favoring the improvement of reproductive parameters such as the number of born animals, lower fetal mortality, and fetal development during gestation.

The NCG molecule activates carbamoylphosphate synthetase, a key enzyme in the process of ARG synthesis in enterocytes, from carbamoyl phosphate and ornithine [15; 16]. In ruminants, dietary NCG supplementation increases the endogenous synthesis of ARG, as NCG is not affected by ruminal metabolic degradation [20]. Thus, NCG supplementation can potentially improve gestation outcomes because of to the increase in ARG compound concentrations in the maternal blood and in uterine fluids [26]. Additionally, NCG does not affect intestinal absorption of amino acids [16].

Up to now, no systematic review regarding the effects of these ARG precursors on fetal development has been reported in the literature. Thus, the objective of this research is to evaluate the effectiveness of CIT and NCG as ARG precursors in mammals and its effects on fetal development through a systematic review.

## Material and methods

### Research strategy

In August 2018, electronic searches were performed in the databases *PubMed*, *ISI Web of Science*, *Science Direct*, *and Scopus* by two independent reviewers (Authors: JYPP and CAPG). The following keywords were used: (arginine precursor OR Citrulline OR N-carbamylglutamate) AND (gestation OR pregnancy OR fetus OR newborn OR reproduction), totaling 15 combinations. The combination of keywords was always two-to-two to ensure that more studies were returned in all databases. During the search, there was no date restriction, and the filter “article type (research articles)” was used according to their availability in the database (Science Direct and Web of Science). This way, books, book chapters, and reviews were excluded.

### Selection of studies

Only studies in which citrulline and N-carbamylglutamate were evaluated in gestating mammals and their effects on some parameters related to the intrauterine development of the concept were selected. Studies with combined supplementation with another amino acids, additives, or medicine were disregarded. When several supplements were tested in the same assay, only the group treated with citrulline and N-carbamylglutamate was considered. When the article evaluated several levels of citrulline or N-carbamylglutamate supplementation, all of these were considered. Papers that evaluated a model of a particular disease were excluded when they did not evaluate any parameters related to the objectives of this review, i.e. reproductive parameters and plasma amino acid concentration.

**Fig 1:**
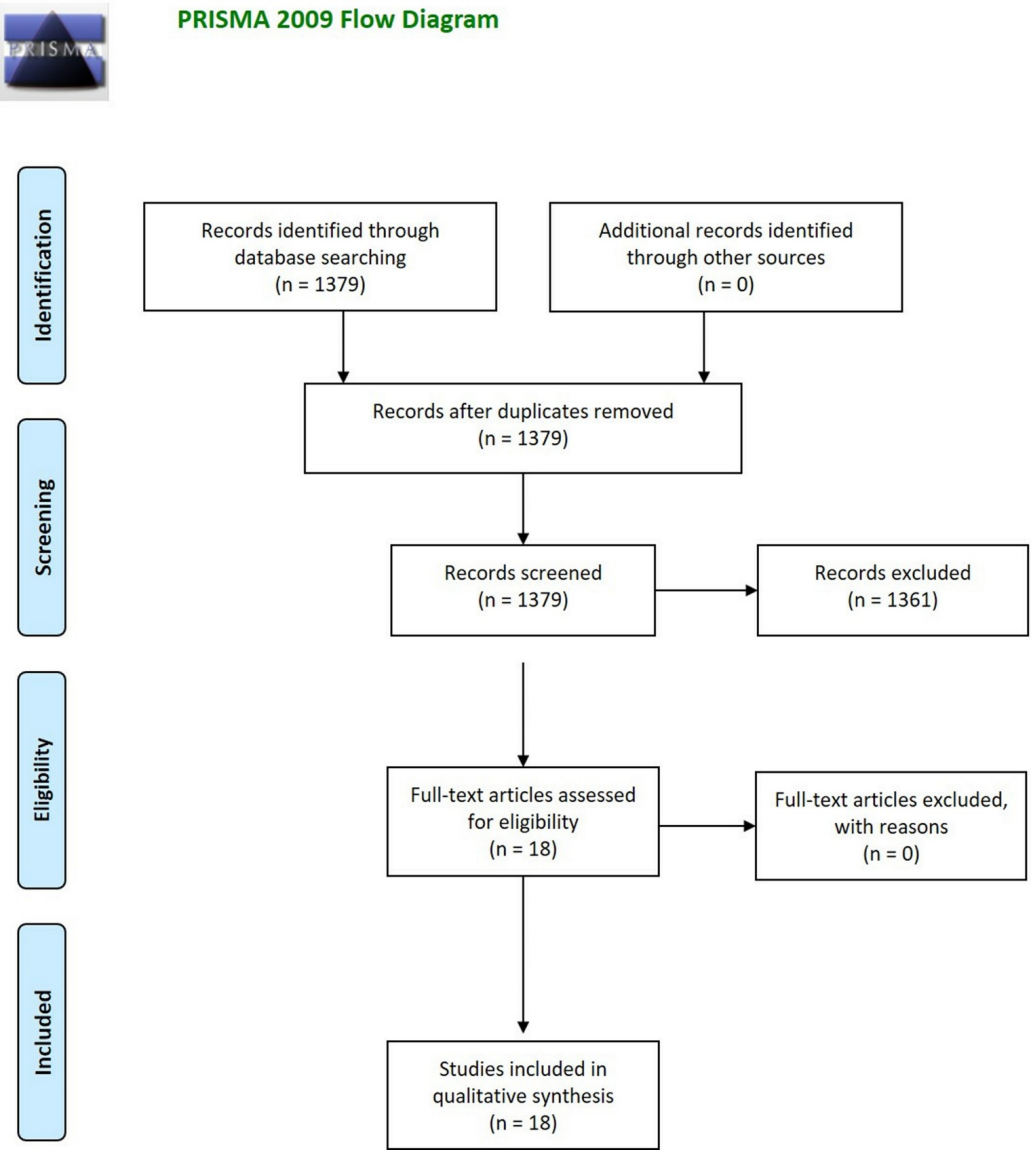
PRISMA diagram. Preferred reporting items for systematic reviews and meta-analysis (PRISMA) flow diagram identifying the total number of articles initially surveyed, the number of articles included and excluded for this systematic review. From: Moher D, Liberati A, Tetzlaff J, Altman DG, The PRISMA Group (2009). Preferred Reporting Items for Systematic Reviews and Meta-Analyses: The PRISMA Statement. PloS Med 6(7): e1000097. https://doi.org/10.1371/journal.pmed.1000097. For more information, visit www.prisma-statement.org.

Of the 1,379 articles found in the databases, 18 articles were selected. Firstly, repeated articles were removed, and the selection was based on the title, abstract, and after considering all content. The researchers carefully ensured that all articles followed the criteria selected for inclusion. In case of discrepancies among the documents, all the criteria were reviewed and discussed among the researchers. Details regarding the search engines are summarized in the preferred reporting items for systematic reviews and meta-analysis (PRISMA) checklist (S1 Table) and in the flow chart (Fig 1).

### Quality criteria

The criteria were adapted based on other systematic reviews [8; 27; 28] and the authors' experience. The following parameters were used:

-Randomization: a randomized study scored 2, while a non-randomized study, or when this was not clearly described in the text, scored 1.
- Blind experiment: a study in which the evaluation was performed by an examiner who was unaware of the evaluated treatments scored 2; when it was not considered or not clearly described in the text, it scored 1.
- Control group: a study that used a control group scored 2, and when it was not present or not mentioned in the text, it scored 1.
- Sample size: when up to 10 experimental units (number of dams) were used per treatment, the study scored 1, and when it used 11 or more, it scored 2.
- Breed or genetic line: a study that mentioned genetic line or breed scored 2, and when it was not mentioned, it scored 1.
- Environment characterization: a study that mentioned environmental parameters scored 2; when they were not mentioned, it scored 1.
-Isonitrogenous diets: studies with isonitrogenous diets scored 2; when not used or mentioned, they scored 1.
- Different dosages: a study that used two or more levels of the precursor scored 2; when only one level was used, the score was 1.
- Parity order: studies that mentioned if the females were controlled scored 2; when this was not mentioned, the studies scored 1.
-Molecular analysis: trials that used molecular analysis to explain the results scored 2; otherwise, they scored 1.

Additional data, such as species, supplementation period, analyzed variables, among others, were used only for descriptive purposes; with no score, just to contribute with the discussion.

## Results

The search results for each database, according to the used keywords and their combinations, are presented in Table 1. The databases that generated the most results were *Science Direct*, *Scopus*, and *Pubmed*. However, most of the articles selected for the present study were initially found in the first used database, *Pubmed*. Only one more article was selected from the *ISI* database. Even with a large number of results, no new articles that fit the selection criteria were added from the databases *Science Direct* and *Scopus*. Finally, out of 1,379 articles, 18 were selected to comprise the study base of the present review.

**Table 1:** Detailed results of the search in each database according to the keywords.

Base	Search	Keywords*			Total
		1	2	3	
**PubMed**	Total number	154	8	44	206
	Selected number	8	9	0	17
	Duplicated number of selected papers	0	0	1	1
**Science Direct**	Total number	229	5	529	763
	Selected number	0	0	0	0
	Duplicated number of selected papers	1	1	1	3
**ISI Web of Science**	Total number	136	9	70	47
	Selected number	0	1	0	0
	Duplicated number of selected papers	8	8	1	17
**Scopus**	Total number	140	8	47	195
	Selected number	0	0	0	0
	Duplicated number of selected papers	8	10	2	20
**Total records identified through database searching**					**1379**
**Number of articles selected for search†**					**18**

^*^1. *Citrulline;*

2. *N-carbamylglutamate;* 3. *Arginine precursor* and its combinations with the following words: *Reproduction*, *gestation*, *pregnancy*, *fetus*, *and newborn*.

^†^Final number of selected articles.

The overall summary information of the selected articles is presented in Table 2. Although the articles were published in the last 12 years, there was no date restriction during the search and selection of studies. From the total number of selected articles, 8 evaluated CIT and 10 evaluated NCG. Considering studies with CIT, the majority (87.5%) used rats, whereas only one study used the goat model. However, in the studies with NCG, 50% used swine, 40% sheep, and only one study used rats. In general, the number of animals varied among the studies, ranging from 3 to 96 animals per treatment.

**Table 2:** Summary of the general data of selected studies to evaluate the effectiveness of arginine precursors during gestation.

#	Author	Species	Control dams	Supplemented dams	Supplementation form	Dosage	Supplementation period	Day of collection	Evaluated parameters
**Citrulline**									
1	Bourdon et al. (2016) [29]	Rat	8 to 10	8 to 10	Water	2 g/Kg BW/day	7 to 21 days of gestation	21 days of gestation	Water and feed intake, maternal weight gain, offspring size and fetal weight, placental weight, fetal and maternal plasma concentration of valine and alanine. Maternal plasma concentration of amino acids. Assessment of tissue protein synthesis; Assessment of urine. Placental gene expression.
2	Tran et al. (2016) [30]	Rat	3 to 4	3 to 4	Water	2 g/Kg BW/day	2–15 and 2–21 of gestation	15 and 21 days of gestation	Feed intake, fetal and placental weight, placental efficiency, placental morphometry, placental gene expression.
3	Tain et al. (2015) [31]	Rat	3	3	Water	2.5 g/L of water	Gestation and lactation	12 weeks of life of male offspring	Weight at 3 months of age, heart and kidney weight, systolic blood pressure; Plasma creatinine concentration, determination of urinary excretion of cyclic guanosine monophosphate; kidney and plasma concentration of amino acids; protein determination, enzymatic activity and gene expression in kidney tissue.
4	Tain et al. (2014a) [21]	Rat	NE	NE	Water	2.5 g/L of water	Gestation and lactation	12 weeks of life of male offspring	Weight at 3 months of age, body weight and organ function parameters; transcriptome and gene expression in kidney tissue.
5	Tain et al. (2014b) [32]	Rat	NE	NE	Water	2.5 g/L of water	Gestation and lactation	16 weeks of age	Blood pressure, plasma concentration of amino acids and metabolites. Expression of proteins and genes in kidney tissue; mortality, live weight, kidney weight.
6	Tain et al. (2010) [33]	Rat	NE	NE	Water	2.5 g/L of water	Gestation and lactation	25, 56 and 84 days after birth and 12 weeks of age	weight, blood pressure, urine collection, renal histology; plasma and tissue concentration of amino acids, enzymatic activity, expression of gene proteins in kidney tissue.
7	Lassala et al. (2009) [34]	Goat	6	6	IV	155 μmol/kg of BW	130 days of gestation	130 days of gestation	Determination of maternal and fetal plasma concentration of amino acids.
8	Koeners et al. (2007) [35]	Rat	6	6	Water	2.5 g/L of water	Gestation and lactation	At birth and two days of age	Concentration of renal and cardiac nitric oxide; renal gene expression; concentration of amino acids: arginine and citrulline; birth weight; blood pressure and renal function.
**N-carbamoyl glutamate**									
9	Cai et al. (2018) [36]	Swine	18	18	Feed	0.5 g/kg of feed	1–8 days of gestation	At birth	Live weight (0 and 28 days of gestation); reproductive performance; blood collection for determination of serum metabolites and amino acids, metabolomic profiles in serum and amniotic fluid; protein abundance in the endometrium, fetuses, and placentae; expression levels of PGRMC1, lamin A/C, eNOS, and vimentin).
9	Cai et al. (2018) [36]	Swine	18	19	Feed	0.5 g/kg of feed	9–28 days of gestation	At birth	Live weight (0 and 28 days of gestation); reproductive performance; blood collection for determination of serum metabolites and amino acids, metabolomic profiles in serum and amniotic fluid; protein abundance in the endometrium, fetuses, and placentae; expression levels of PGRMC1, lamin A/C, eNOS, and vimentin).
9	Cai et al. (2018) [36]	Swine	18	17	Feed	0.5 g/kg of feed	1–28 days of gestation	At birth	Live weight (0 and 28 days of gestation); reproductive performance; blood collection for determination of serum metabolites and amino acids, metabolomic profiles in serum and amniotic fluid; protein abundance in the endometrium, fetuses, and placentae; expression levels of PGRMC1, lamin A/C, eNOS, and vimentin).
10	Sun et al. (2018) [37]	Sheep	8	8	Feed	5 g/day	35–110 days of gestation	110 days of gestation	Female and fetal weight; fetal organ weight; fetal plasma metabolite and hormone concentrations; amino acid concentrations in the fetal liver, amino acid concentrations of fetal longissimus dorsi muscle and expression of genes of the somatotropic axis.
11	Sun et al. (2017) [14]	Sheep	8	8	Feed	5 g / day	35–110 days of gestation	110 days of gestation	Female weight, fetal, and placentome weight and magnetic resonance of the blood plasma for assessment of 36 metabolites.
12	Zhang et al. (2016a) [38]	Sheep	8	8	Feed	5 g/day	35–110 days of gestation	110 days of gestation	Female weight, maternal organ weight, fetal organ weight and placentome size; serum concentrations of metabolites and hormones; amino acid concentrations in maternal artery, fetal umbilical vein, and in fetal fluids; concentration of polyamines in maternal, fetal, and placental fluids.
13	Zhang et al. (2016b) [39]	Sheep	8	8	Feed	5 g/day	35–110 days of gestation	110 days of gestation	Fetal weight; type, number, and average weight of placentomes; total weight and number of placentomes; antioxidant capacity in the maternal and fetal plasma and in the types of placentomes; concentration of mRNA of selected angiogenic and vasoactive factors and their receptors in placentomes; concentrations of metabolites and hormones.
14	Zhu et al. (2015) [40]	Swine	16	16	Feed	1.1 g/day	1 to 28 days of gestation	28 days of gestation	Maternal weight, number of total and living fetuses; number of corpora lutea; embryonic mortality; uterine weight; weight of viable fetuses and placenta, volume of amniotic fluid; glutamate, ornithine, arginine, and proline concentrations, NO, estradiol and progesterone in maternal plasma at 14 and 28 days of age; protein and gene expression of the endometrium.
15	Zhang et al. (2014) [15]	Swine	9	9/9/9/9	Feed	0.5, 1.0, 1.5, and 2.0 g/kg of feed	Throughout gestation	Birth	Live weight and backfat thickness (0, 30 and 110 days of gestation); blood collection for determination of amino acids, angiogenic factors and metabolites (30, 60, 90, and 110 days of gestation); weight of piglets at birth, live-born, stillbirths, and mummified, placental weight. chorioallantoic membrane sample for evaluation of gene expression (at birth).
16	Liu et al. (2012) [41]	Swine	9	9	Feed	1 g/kg of feed	Throughout gestation	110 days of gestation and at birth	Number of births, birth weight, individual birth weight, classification as live-born, stillbirth, or mummified, plasma concentration of hormones, metabolites, and amino acids. Gene expression in the umbilical vein.
17	Zeng et al. (2012) [26]	Rats	12	12	Feed	1 g/kg of feed	Day 1 to 4 of gestation	Day 7 and 15	Administration of antibody LIF (mTOR inhibitor); assessment of fetal weight, concept, placenta, and number of live fetuses
17	Zeng et al. (2012) [26]	Rats	15	15	Feed	1 g/kg of feed	Day 1 to 4 of gestation	Day 5 (slaughter)	Blood and uterine fluid to assess concentrations of amino acids and metabolites.
17	Zeng et al. (2012) [26]	Rats	96	96/96	Feed	0.5 and 1 g/kg of feed	Throughout gestation	Birth	Number of births, birth weight, individual birth weight, mortality, and sex.
18	Wu et al. (2012) [23]	Swine	9	9	Feed	1 g/kg of feed	Throughout gestation	110 days of gestation and at birth	Blood for analysis of metabolites, minerals, hormones, and amino acids. Number of births, birth weight, live-born. Gene expression of the umbilical vein (at birth).

**NE**: unspecified; **IV**: intravenous

Regarding the form of supplementation, CIT was supplemented in most of the studies (87.5%) via water, while NCG supplementation was in the form of a feed additive in all studies. For CIT, the dose used was 2.5 g/L of water in most studies (62.5%). However, two studies used 2 g per kg of live weight per day [29; 30], and Lassala et al. [34] supplemented 155 micromoles per kilogram of BW per day for goats. For NCG, the supplementation varied from 0.5 to 5 g per kg of ration; the dose used in 40% of the studies was 1 g per kg of ration. Four studies supplemented 5 g per day for sheep, one study with 1.1 g per day for swine and one study with 0.5 g per day for swine. Regarding the supplementation period, all studies with CIT supplemented throughout the gestation period; two of which included evaluations up to 7 and 15 days of gestation in rats. Of the studies with NCG, 40% supplemented throughout gestation, while another 40% of the studies with sheep supplemented from 35 to 110 days of gestation. two of the studies with pigs supplemented in the initial third of gestation, within the first 28 days.

Several variables were evaluated in the studies, including those related to the animal’s reproductive performance, progeny performance, concentrations of hormones and metabolites in the blood, concentrations of amino acids in plasma and other tissues, and gene expression (Table 2).

The quality of the selected articles was evaluated considering some previously established criteria (Table 3). As all the papers presented the same score for the criteria “blind experiment”, “breed or genetic line”, and “animal ethics committee”, these were disregarded for the score. The maximum score achieved was 14 points [26] out of 18 possible points, while the minimum score was 9 points [35; 34]. Of all articles, the majority (66.67%) described that the studies were randomized. No article was reported as a blind experiment of the studied variables. Most studies used a control group (88.89%), while the sample size was below 10 replicates per treatment in most studies (83.33%).

**Table 3:** Quality of the selected articles considering the selected criteria.

Author/Year	A	B	C	D	E	F	G	H	Total
**Citrulline**									
Bourdon et al. (2016) [29]	2	2	1	2	2	1	1	2	**13**
Tran et al. (2016) [30]	2	2	1	2	1	1	1	2	**12**
Tain et al. (2015) [31]	1	2	1	1	1	1	2	2	**11**
Tain et al. (2014a) [21]	2	2	1	1	1	1	2	2	**12**
Tain et al. (2014b) [32]	1	2	1	1	1	1	2	2	**11**
Tain et al. (2010) [33]	1	2	1	1	1	1	2	2	**11**
Lassala et al. (2009) [34]	1	1	1	1	1	1	2	1	**9**
Koeners et al. (2007) [35]	1	1	1	1	1	1	1	2	**9**
**N-carbamylglutamate**									
Cai et al. (2018) [36]	2	2	2	1	1	1	2	2	**13**
Sun et al. (2018) [37]	2	2	1	1	1	1	2	2	**12**
Sun et al. (2017) [14]	2	2	1	1	1	1	2	1	**11**
Zhang et al. (2016a) [38]	2	2	1	2	1	1	2	1	**12**
Zhang et al. (2016b) [39]	2	2	1	2	1	1	2	2	**13**
Zhu et al. (2015) [40]	2	2	2	1	1	1	2	2	**13**
Zhang et al. (2014) [15]	1	2	1	1	1	2	2	2	**12**
Liu et al. (2012) [41]	2	2	1	1	1	1	2	1	**11**
Zeng et al. (2012) [26]	2	2	2	2	1	2	1	2	**14**
Wu et al. (2012) [23]	2	2	1	1	1	1	2	2	**12**

**A**. Randomization: 2 for randomized studies and 1 for non-randomized studies or no clarification of this aspect in the text;

**B**. Control group: 2 for studies that used a control group and 1 for studies that used no control group or did not clarify this in the text;

**C**. Sample size: 2 for studies that used more than 10 replicates per treatment and 1 for studies that used 10 or less replicates per treatment;

**D**. Environment characterization: 2 for studies that evaluated environmental parameters and 1 when this was not mentioned or not clear at the text;

**E**. Isonitrogenous diets: 2 for studies that used isonitrogenous diets and 1 for studies that did not use such diets are did not specify this in the text;

**F.** Different dosages: 2 for studies that used two or more levels of the precursor and 1 for studies that used only one level;

**G**. Parity order: 2 for studies that mentioned if the parity order of the dams was controlled and 1 for studies that did not mention this;

**H.** Molecular analysis: 2 for trials that used molecular analysis to explain the results and 1 for studies that did not use such analyses.

Only 27.8% of the studies [29; 30; 26; 38; 39] evaluated some environmental variables during the experiment. Only one study [29] used isonitrogenous diets, and some studies (11.1%) used two or more levels of the precursor [15; 26]. The majority of the studies (77.8%) mentioned the parity order of the females and used molecular analysis to explain the results.

The main reproductive performance results obtained using CIT and NCG as arginine precursors for gestating mammal, as well as general considerations regarding these results, are presented in Table 4. All data were compared to the control as a ratio, dividing one value per another and multiplying per 100. Supplementation of CIT as an arginine precursor did not influence the placental weight in the three trials that evaluated this variable. By supplementing NCG, significant effects were found in 40% of the studies that evaluated this variable.

**Table 4:** Supplementation effects of citrulline and N-carbamylglutamate for gestating mammals, compared to the control group, on reproductive performance parameters.

Author/Year	Dose	Placental	Fetuses	Live fetuses	Weight fetuses	Weight of viable fetuses	Mortality	Benefits
**Citrulline**								
Bourdon et al. (2016) [29]	2 g/kg of BW	NS	NS	NE	8.64%	NE	NE	YES
Tran et al. (2016) (Day 15) [30]	2 g/ kg of BW	NS	NE	NE	NS	NE	NE	IND
Tran et al. (2016) (Day 21) [30]	2 g/ kg of BW	NS	NE	NE	4.00%	NE	NE	YES
Tain et al. (2015) [31]	2.5 g/L of water	NE	NS	NE	NS	NE	NS	IND
Tain et al. (2014a) [21]	2.5 g/L of water	NE	NS	NE	NS	NE	NS	IND
Tain et al. (2014b) [32]	2.5 g/L of water	NE	NE	NE	NE	NE	NE	NE
Tain et al. (2010) [33]	2.5 g/L of water	NE	NE	NE	NE	NE	NE	NE
Lassala et al. (2009) [34]	2.5 g/L of water	NE	NE	NE	NE	NE	NE	NE
Koerners et al. (2007) [35]	155 micromol/kg of BW	NE	NE	NE	NE	NE	NE	NE
**N-carbamylglutamate**								
Cai et al. (2018) [36]	0.5 g/kg (1–8 days)	NE	NS	NS	NE	NS	NS	IND
	0.5 g/kg (9–28 days)	NE	NS	8.12%	NE	NS	NS	YES
	0.5 g/kg (1–28 days)	NE	9.63%	11.34%	NE	NS	NS	YES
Sun et al. (2018) [37]	5g/day	NE	NE	NE	NE	↑	NE	YES
Sun et al. (2017) [14]	5g/day	NS	NE	NE	20.00%	NE	NE	YES
Zhang et al. (2016a) [38]	5g/day	NS	NE	NE	20.10%	NE	NE	YES
Zhang et al. (2016b) [39]	5g/day	NS	NE	NE	19.15%	NE	NE	YES
Zhu et al. (2015) [40]	1.1 g/day	12.87%	11.50%	11.82%	NE	17.98%	-51.53%	YES
Zhang et al. (2014) [15]	0.5 g/kg of ration	14.62%	NS	NS	6.57%	19.17%	NS	YES
Liu et al. (2012) [41]	1 g/kg of ration	NE	NS	NS	NS	13.60%	-61.11%	YES
Zeng et al. (2012) [26]	1 g/kg of ration	NE	6.90%	7.02%	NS	6.75%	NE	YES
	0.5 g/kg of ration	NE	12.93%	14.04%	NS	14.05%	NE	YES
Wu et al. (2012) [23]	1 g/kg of ration	NE	NS	NS	8.90%	NE	NE	YES

**NS**: not significant; **NE**: not evaluated; **IND**: indifferent; ↑: increase

Supplementation of CIT did not significantly affect the number of fetuses or total births. However, 50% of the NCG studies that evaluated these variables showed positive effects, increasing the number of fetuses or total births. The number of fetuses or live-born animals was measured in nine studies that supplemented NCG; five of these studies verified an increase in these variables.

Fetus weight and number of total births were influenced in 40% of the studies with CIT that evaluated this variable. The majority (62.5%) of the studies that supplemented NCG found increased fetal or total birth weight; N-carbamoyl glutamate supplementation improved the weight of fetuses or live-borns in most trials (66.67%) that evaluated this variable.

The mortality of embryos, fetuses, or newborns was not affected in studies evaluating CIT; however, when NCG was supplemented, this variable decreased in relation to the control group in two studies [40; 41].

Overall, supplementation of CIT for gestating mammals resulted in reproductive performance benefits in only 22.22% of the studies. In the other studies, 33.33% found no benefits of the supplementation of this amino acid (not statistically significant), and 44.44% of the trials did not evaluate reproductive parameters. An NCG supplementation during gestation had at least one positive effect on the variables of reproductive performance in 93.31% of the studies; only one study reported not statistically significant results when NCG was supplemented within the first 28 days of sow gestation.

The effects of CIT and NCG supplementation on plasmatic or tissue amino acid concentrations are presented in Table 5. All data were compared to the control as a ratio, dividing one value per another and multiplying per 100. By supplementing CIT, 40% of the studies reported an increase in arginine maternal plasma concentrations, while 33% of the studies reported increased concentrations of ornithine and CIT. Plasma concentrations of other amino acids were not influenced by CIT supplementation.

**Table 5:** Supplementation effects of citrulline and N-carbamylglutamate for gestating mammals on plasmatic or tissue amino acid concentrations.

Reference	Evaluated material	Amino acids*											
		Arginine	Proline	Ornithine	Citrulline	Aspartate	Glutamate	Glutamine	Alanine	NCG	ADMA	SDMA	Profiles of other AAs†
**Citrulline**													
Bourdon et al. (2016) [29]	Plasmatic	115.00%	NE	280.00%	360.00%	NE	NE	NE	NE	NE	NE	NE	NE
Tran et al. (2016) [30]	NE	NE	NE	NE	NE	NE	NE	NE	NE	NE	NE	NE	NE
Tain et al. (2015) [31]	Plasmatic	NS	NE	NE	NS	NE	NE	NE	NE	NE	NS	NS	NE
Tain et al. (2014a) [21]	NE	NE	NE	NE	NE	NE	NE	NE	NE	NE	NE	NE	NE
Tain et al. (2014b) [32]	NE	NS	NE	NE	NS	NE	NE	NE	NE	NE	NS	NS	NE
Tain et al. (2010) [33]	Plasmatic	31.00%	NE	NE	NS	NE	NE	NE	NE	NE	-3.33%	3.77%	NE
Lassala et al. (2009) [34]	Maternal	↑	NE	NS	↑	NE	NE	NE	NE	NE	NE	NE	NE
	Fetal	↑	↑	↑	↑	NE	NE	NE	NE	NE	NE	NE	NE
Koerners et al. (2007) [35]	Female—heart	1.15%	NE	NS	NS	NE	NE	NE	NE	NE	NE	NE	NS
	Female—kidney	NS	NE	NS	NS	NE	NE	NE	NE	NE	NE	NE	NS
	Male—heart	-5.06%	NE	-19.35%	NS	NE	NE	NE	NE	NE	NE	NE	NS
	Male—kidney	NS	NE	NS	NS	NE	NE	NE	NE	NE	NE	NE	NS
**N- carbamylglutamate**													
Cai et al. (2018) [36]	NE	NE	NE	NE	NE	NE	NE	NE	NE	NE	NE	NE	NE
Sun et al. (2018) [37]	Fetal liver	NS	45.23%	26.84%	24.70%	37.72%	26.38%	12.97%	NS	NE	NE	NE	YES †††
	Fetal *Longissimus dorsi* muscle	72.51%	NS	NS	11.19%	33.89%	21.95%	15.40%	18.81%	NE	NE	NE	YES ††††
Sun et al. (2017) [14]	NE	NE	NE	NE	NE	NE	NE	NE	NE	NE	NE	NE	NE
Zhang et al. (2016a) [38]	Plasmatic	50.84%	52.24%	44.00%	32.24%	46.34%	NS	NS	46.40%	NE	NE	NE	YES ††
Zhang et al. (2016b) [39]	Plasmatic	NE	NE	NE	NE	NE	NE	NE	NE	NE	NE	NE	NE
Zhu et al. (2015) [40]	Plasmatic	30.21%	14.44%	43.33%	NE	NE	16.86%	NE	NE	NE	NE	NE	NE
		38.97%	17.20%	23.85%	NE	NE	41.18%	NE	NE	NE	NE	NE	NE
Zhang et al. (2014) [15]	Plasmatic	11.97%	-7.35%	13.29%	NE	NE	NE	NE	NE	NE	NE	NE	NE
	Plasmatic	8.99%	-7.79%	14.86%	NE	NE	NE	NE	NE	NE	NE	NE	NE
	Plasmatic	8.64%	-8.56%	17.80%	NE	NE	NE	NE	NE	NE	NE	NE	NE
	Plasmatic	NS	-8.33%	15.05%	NE	NE	NE	NE	NE	NE	NE	NE	NE
Liu et al. (2012) [41]	Plasmatic	40.63%	-46.67%	NE	NE	5%	NE	NS	NE	NE	NE	NE	NE
Wu et al. (2012) [26]	NE	NE	NE	NE	NE	NE	NE	NE	NE	NE	NE	NE	NE
Zeng et al. (2012) [23]	Plasmatic	49.16%	20.36%	69.66%	NE	NE	NS	NS	NE	NE	NE	NE	NE
	Uterine fluid	18.77%	139.35%	NS	NE	NE	34.15%	49.56%	NE	NE	NE	NE	NE

**SDMA:** symmetric dimethylarginine;

**ADMA:** asymmetric dimethylarginine;

*AA concentration increase compared to the control group;

**†** Profile change of the other AA;

**††** Changed: Methionine (50.00%), Isoleucine (30.16%), Leucine (33.33%), Cysteine (38.30%).

**†††** Changed: Lysine (23.30%), Methionine (63.14%), Phenylalanine (31.28%), Threonine (43.08%), Tryptophan (37.24%), Tyrosine (54.28%).

**††††.** Changed: Isoleucine (21.70%), Leucine (28.70%), Lysine (18.94), Methionine (35.58%), Phenylalanine (42.90%), Threonine (28.72%), Valine (24.76%), Asparagine (30.67%), Glycine (-51.36%).

**NS**: not significant; **NE**: not evaluated; **↑:** increase

The NCG supplementation increased maternal plasma concentrations of arginine in all studies that evaluated this variable. Plasma concentrations of proline were increased in 60% of the studies, while 40% recorded a decrease.

Plasma concentrations of the amino acids citrulline, ornithine, aspartate, and alanine increased in all studies evaluating these amino acids, while glutamate concentrations increased only in one study [40]. Plasma concentrations of the other amino acids were only altered in one of the studies [26].

## Discussion

Systematic reviews provide a broad view on study results from a given theme over the years, generating comprehensive conclusions, new knowledge, and starting points for new lines of research.

Our goal was to survey all papers involved in direct precursors of L-arginine, with the aim to show other possible effects to be explored. We started with a pre-review to assess the state of the art in this specific subject, and the results obtained in this previous survey led us to decide to delimit our search specifically for the two precursors presented in this review (CIT and NCG), mainly because the numbers of studies for other precursors were limited, making it impossible to infer any results about their effectiveness. Some precursors such as glutamate and glutamine are not directly precursors exclusive to L-arginine and its supplementation aims other metabolic pathways to improve reproduction and fetal development, and because of that were not included in this revision.

The criteria to verify the quality of the selected articles were the presence or absence of randomization, blind experiment, control group, and number of animals per treatment. As part of the results, no study was indicated as being a blind experiment, which is considered essential since it discards any kind of bias during the procedures, providing increased credibility to the results [42].

Sample size is another important aspect, i.e. the number of animals used per treatment. Most studies used less than 10 animals per treatment. Although there is no minimum standard number, it is clear that the greater the experimental units, i.e., repetitions, the smaller the standard errors of the treatment mean, resulting in a greater reliability of experiment treatment differences. However, factors such as species, response variables, availability of facilities, animal welfare, and even financial resources limit the use of more representative samples.

The relevance of temperature in animal production and research is increasingly being discussed and considered as an aspect extremely important due to its direct relationship with animal performance; in this sense, the characterization of the thermal environment where the animals are kept is fundamental [43]. In the present review, the environment was measured or monitored in less than a third of the studies. This aspect becomes even more relevant in nutrition studies, since environmental conditions may interfere with the absorption and metabolic fate of nutrients; in relation to ARG, blood flow can be redirected to the peripheral organs under heat stress situations, resulting in an increased demand for this amino acid [44; 45].

Considering the nutritional composition, the use of isonitrogenous diets is also important, especially in studies that evaluate the effects of amino acids since the total nitrogen concentration of the diet could be a source of variation among the treatments. In the present review, only one article used isonitrogenous diets [29].

Finally, the use of molecular analyses to explain the results is important as it can help to explain the effects of a certain nutritional strategy, including mainly the metabolic pathways by which these effects happen, offering a greater certainty of the results found.

With the objective of reducing the occurrence of lightweight animals at birth in mammalian species, nutritional studies have proposed the use of functional nutrients that favor fetal development during gestation. In this sense, the use of functional amino acids has been widely discussed in recent years [5; 46]. This includes the application of ARG, which is considered a conditionally essential amino acid in the diet for some animal categories and necessary to meet specific animal body needs [5] associated with important functions in the reproductive processes, especially during the gestation of mammals, where it plays an essential role in fetal development.

Arginine is recognized as a precursor for the synthesis of several important metabolic molecules, including nitric oxide and polyamines, which actively participate in fetal development [5; 47]. Nitric oxide has been associated with increased blood flow [48], while polyamines stimulate angiogenesis, embryogenesis, and placental growth [49]. In addition, this amino acid has functions in the regulation of gene expression, cell signaling, and enzymatic activity [46], and its oral administration is considered safe for animals [18].

**Fig 2:**
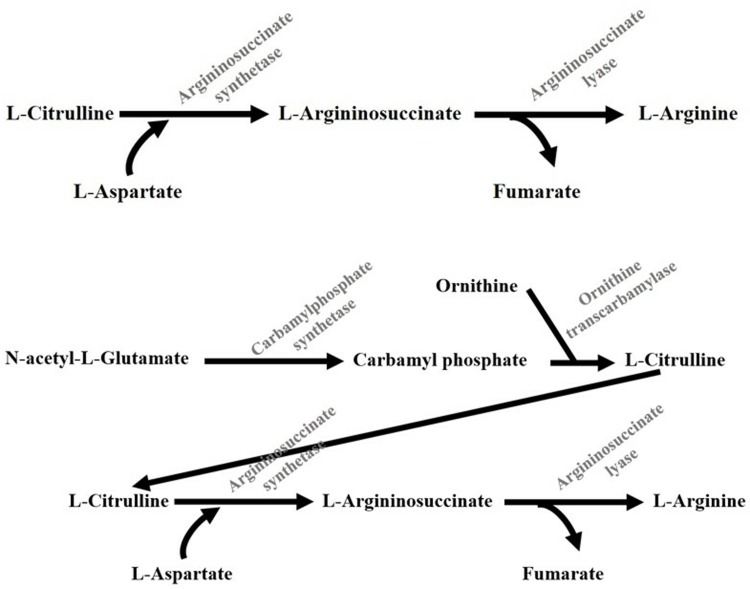
Metabolic pathways of arginine precursors conversion to arginine. Adapted from [53].

In the present study, the use of ARGs in pigs, sheep, and rats has shown the effectiveness of ARG in terms of fetal development [26; 50; 51]. However, ARG supplementation has some limitations, primarily related to its rapid *in vivo* metabolism, high cost, and controversial results for some species [8; 15; 52]. In this context, the use of precursors for endogenous arginine synthesis could be a promising route to explore the important reproductive functions of this amino acid, associated with its economic viability in mammalian species. Both NCG and CIT have been proposed as arginine precursors, and the supplementation of these compounds in the diet of gestating mammals could increase ARG biosynthesis (Fig 2).

The NCG molecule is a structural analogue of N-acetylglutamate, which activates carbamoyl phosphate synthetase-1, a key enzyme in the arginine biosynthesis pathway [16]. Thus, NCG supplementation favors the endogenous synthesis of ARG, increasing the availability of this amino acid in the animal organism [54; 55].

Based on the selected studies, NCG supplementation had in positive effects on mammalian reproductive performance in all articles, mainly influencing fetal development with increased live birth weight and, in some situations, increased number of live-borns, lower embryo mortality, and increased placental weight. In contrast, CIT supplementation was not as effective in improving reproductive characteristics.

In gestating mammals, NCG supplementation was effective in increasing ARG concentrations in the maternal circulation and in uterine fluids, favoring greater fetal development [26]. Additionally, NCG is not extensively degraded in the rumen of ruminant animals, which would favor its subsequent absorption and effects [20].

In the present review, NCG supplementation increased maternal plasma concentrations of ARG in all studies that evaluated this variable and, in some cases, other amino acids in the ARG family increased their concentrations. These results lead us to infer that NCG supplementation has effectively provided more ARG in the animal organism, which supports the effects on placental and fetal development, also presented in this review.

In recent studies, researchers report the first evidence of other pathways by which NCG supplementation could influence fetal development. For example, Zhu et al. [40] evidenced alterations in the endometrial proteome by supplementing NCG for pregnant gilts and verified differences in the expression of 59 proteins in the supplemented group, which are mainly involved in cell adhesion, energy metabolism, lipid metabolism, protein metabolism, oxidative stress, and immune response.

The CIT molecule is an important precursor of ARG in the animal organism. This amino acid escapes from the hepatic metabolism and appears in the blood to be absorbed into the kidneys and converted to arginine via the enzymes argininosuccinate synthase (ASS) and argininosuccinate lyase (ASL) [12; 24]. Furthermore, CIT has a limited degradation in the placenta, being efficiently transferred from the mother to the fetus [5].

Besides its beneficial effects on reproductive characteristics, another important aspect to be considered is that CIT supplementation has been associated with improved renal function and therefore used as an alternative to prevent hypertension [21; 30; 31; 32; 35]. During fetal development, the kidney is particularly susceptible to nitric oxide deficiency. Deficiencies of this metabolite during gestation alter renal function, leading to hypertension in adulthood [56; 57]. In this context, maternal supplementation of CIT aims to increase the bioavailability of nitric oxide via ARG in order to prevent hypertension. Thus, most of the studies that evaluated CIT supplementation in the present review sought to explore this pathway of nitric oxide, assessing renal gene expression and its relationship with disease prevention [21; 30; 31; 32; 35].

Regarding supplementation efficacy, the study of Lassala et al. [34] showed that intravenous administration of L-citrulline in pregnant sheep resulted in greater concentrations of ARG in maternal and fetal plasma in relation to L-arginine administration. Other studies have shown that supplementation of L-citrulline in pregnant rats increased fetal weight and placental efficiency [29] as well as increased the expression of angiogenic genes and placental growth factors [30].

Therefore, CIT supplementation could be an alternative approach to increase the availability of ARG during gestation, thus favoring fetal development; however, in most of the selected studies, an increase in ARG concentration and benefits on reproductive performance were not evidenced.

In the present review, CIT supplementation had no significant effects on fetal development in most studies evaluating reproductive variables (60%); only two studies showed an increase in live birth weight [29; 30].

The lack of positive results obtained with CIT supplementation could be related to the animal species in which this amino acid was tested, since the majority (87.5%) of the CIT studies were performed with rats. The form and level of the used supplementation may also interfere with the actual intake and use of this amino acid. In this case, the form of CIT supplementation in the studies with rats presented in this review was via water, in contrast to all other studies with NCG supplementation performed via the ration.

Arginine precursors have different tissue-specific effects. The CIT is mainly converted into L-arginine in the kidneys [58], and therefore, CIT supplementation in mammals with kidney disease might cause less L-citrulline-to-L-arginine conversion. In a different way, NCG is mainly converted in ARG in the gut and the liver [59].

The period of supplementation is another factor that could affect the results. For this reason, we divided the supplementation into three periods, initial, intermediate, and final third of gestation. For the different species, the first third of gestation is general linked to the formation of the primary muscle fibers, while in the final third, the fetuses show the greatest growth [60; 61; 62]. For CIT, most of the trials supplemented during the entire period, while for NCG, some supplemented ion the first third or for the entire gestation period, but in contrast to CIT supplementation, NCG was more effective in increasing the weight of the offspring, irrespective of the supplementation period.

Some effects of CIT or NCG could be more pronounced if the dams were subjected to a pathology or challenge such as defects in NO signaling, which some of the weaker offspring may experience, or a greater number of fetuses are being gestated. As described by [63], the authors found no or only a small effect on fetal weight in healthy pregnancies with the supplementation of sildenafil in pregnant animals.

Comparing the effects of ARG supplementation with those of its precursors (CIT or NCG) on fetal development, the effects of NCG supplementation are equivalent to those of ARG. This has been observed in sheep [14; 37; 38; 39] and in pigs [23; 26]. However, studies that evaluated the supplementation of ARG and CIT in the same experimental conditions are limited. Only one trial found similar effects in rats [29]. Lassala et al. [34] concluded that intravenous administration of citrulline is more effective than that of arginine in sustaining high concentrations of arginine in the maternal and fetal circulations of pregnant ewes. Generally, considering animal production systems, factors such as the cost of the supplement, availability, and the economic return should be considered when making a decision. For humans, supplementation with these precursors may be a viable alternative, guaranteeing the best development of the fetus by greater availability of nutrients.

The efficacy of NCG supplementation during gestation to improve mammalian reproductive efficiency and fetal development was documented in the present review. The potential of this supplement is demonstrated for future research evaluating inclusion levels and supplementation periods in mammalian species, aiming at its technical and economic viability in livestock production systems. In relation to CIT, new research should be performed in other animal species exploring other forms of supply, with the objective of evaluating the supplementation effects of this amino acid on the reproductive performance of mammals. The overall results of this review also provide possibilities for research with the use of these precursors in studies with humans.

## Conclusions

Our findings suggest that the arginine precursor N-carbamoyl is more effective than L-citrulline during the gestation of mammals. For this reason, we recommend evaluation of both precursors to be evaluated in research trial with an L-Arginine treatment to confirm existence compound specific effects. The trials should include period and levels of supplementation fed and consider different animal species. The supplementation of NCG increases arginine concentrations in maternal plasma, which improves mammalian reproductive efficiency and fetal development, mainly promoting higher birth weight.

## Supporting information

S1 Table PRISMA checklist. The preferred reporting items for systematic reviews and metanalysis (PRISMA) checklist reflects 27 items under seven main categories that highlight the essential components of this systematic review.
(DOC)
